# The coexistence of hypercalcemia, osteoporosis and thymic enlargement in graves’ disease: a case report

**DOI:** 10.1186/s12902-020-00583-8

**Published:** 2020-06-30

**Authors:** Dandan Yan, Yanjun Xu, Lian-Xi Li

**Affiliations:** 1 Department of Endocrinology and Metabolism, Shanghai Jiao Tong University Affiliated Sixth People’s Hospital, Shanghai Diabetes Institute, Shanghai Clinical Center for Diabetes, Shanghai key Laboratory of Diabetes Mellitus, 600 Yishan Road, Shanghai, 200233 China; 2 Department of Ultrasound in Medicine, Shanghai Jiao Tong University Affiliated Sixth People’s Hospital, Shanghai Institute of Ultrasound in Medicine, 600 Yishan Road, Shanghai, 200233 China

**Keywords:** Graves’ disease, Hypercalcemia, Osteoporosis, Thymic enlargement, Case report

## Abstract

**Background:**

Hyperthyroidism-induced hypercalcemia has been reported previously, but hypercalcemia accompanied by severe osteoporosis and significant thymic enlargement in patients with hyperthyroidism is quite rare. We report the coexistence of hypercalcemia, osteoporosis and thymic enlargement in a patient with Graves’ disease.

**Case presentation:**

A 22-year-old female was diagnosed as Graves’ disease with obviously elevated serum calcium and reduced parathyroid hormone levels. Dual-energy x-ray absorptiometry and chest enhanced computer tomography (CT) revealed severe osteoporosis and a significant enlargement of thymus. After the successful control of hyperthyroidism with methimazole, hypercalcemia was corrected, bone mineral density was improved and thymus also shrank obviously.

**Conclusion:**

This is a very rare case of hypercalcemia accompanied by severe osteoporosis and significant thymic enlargement induced by Graves’ disease. In clinical practice, examination of thymus and bone density should be considered when a patient with Graves’ disease was present with hypercalcemia.

## Background

As a common reason of hyperthyroidism, Graves’ disease may be associated with other autoimmune disorders [1], among which thymic hyperplasia was first described in 1912 and had been reported repeatedly thereafter [2, 3]. However, thymic enlargement is relatively rare in patients with Graves’ disease according to previous studies [4, 5]. Additionally, patients with Graves’ disease can be manifested as hypercalcemia, hypercalcemia crisis even osteoporosis except for the common clinical features [6, 7]. Furthermore, Graves’ disease accompanied by hypercalcemia and thymus enlargement had previously been described only in one case report [8].

Here, we presented a very rare case of hypercalcemia and severe osteoporosis induced by Graves’ disease accompanied with remarkably thymic enlargement simultaneously. We summarized the clinical procedure of this patient, which will be meaningful for the clinical diagnosis and treatment of such a condition.

## Case presentation

A 22-year-old female was admitted to our Department of Endocrinology and Metabolism on July 26, 2018. One month prior, she developed symptoms of heat intolerance, increased sweating, palpitation, and polyphagia, and was diagnosed as Graves’ disease based on increased levels of free triiodothyronine (FT3), free thyroxine (FT4) and anti-thyrotropin- receptor antibody (TRAb), and suppressed thyroid stimulating hormone (TSH). The local physician gave her methimazole 10 mg, 3 times a day and metoprolol sustained-release tablets 47.5 mg, 1 time a day. However, the therapy was discontinued 4 days later due to elevated alanine aminotransferase (107 U/L, normal range: 0–65 U/L), so she was referred to our department to treat hyperthyroidism and liver dysfunction. There was no family history of thyroid disease, hypercalcemia and malignancy.

**Fig. 1:**
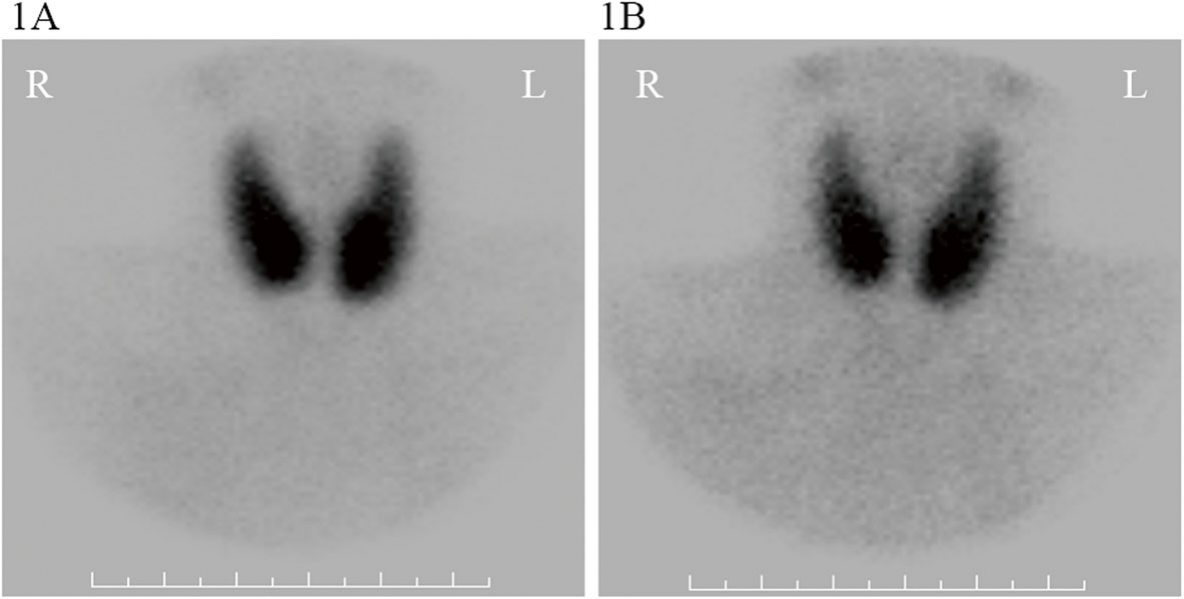
Parathyroid emission computerized tomography (ECT) scan of the patient. It demonstrated diffuse enlargement thyroid with high uptake of 99 m-Tc at both the early phase (**a**) and the delayed phase (**b**), and no parathyroid lesions with high uptake of 99 m-Tc were found in both phases

On admission, the patient complained of hyperthyroid symptoms such as heat intolerance, increased sweating and palpitation, and physical examination showed a markedly enlarged thyroid gland. The ultrasound examination revealed diffuse enlargement of thyroid with abundant blood flow. The emission computerized tomography (ECT) scan demonstrated diffuse enlargement thyroid with high uptake of 99 m-Tc, and no parathyroid lesions with high uptake of 99 m-Tc were found in early and delayed phases (Fig. 1). Laboratory examination showed that TSH was 0.01 mU/L (normal range: 0.27–4.2 mU/L), FT3 > 50 pmmol/L (normal range: 3.1–6.8 pmmol/L), FT4 > 100pmmol/L (normal range: 12–22 pmmol/L), and TRAb was 31.07 U/L (normal range: 0–1.58 U/L). The clinical features combined with laboratory and auxiliary examination indicated a diagnosis of Graves’ disease. So we treated her with polyene phosphatidyl choline 456 mg, 3 times a day and bicyclol 25 mg, 3 times a day for liver protection, propranolol 20 mg, 4 times a day for heart rate control. It was hard to identify whether methimazole or hyperthyroidism caused elevated alanine aminotransferase, so we tried a small dose of methimazole (10 mg 1 time a day) to correct hyperthyroidism after her liver damage recovered and kept monitoring her liver function. The liver damage recovered soon and remained in the normal range, but the patient developed new symptoms of nausea and vomit, and the electrolyte examination showed an obviously elevated blood calcium level (3.22 mmol/L).

Therefore, further examinations were performed to investigate the causes of hypercalcemia. The parathyroid hormone (PTH) was low (3.16 ng/L, normal range: 15–65 pg/ml). Primary hyperparathyroidism with normal or low PTH has been reported although they are relatively rare [9, 10], so we performed a 99mTc-MIBI scintigraphy to exclude this diagnosis. 99mTc-MIBI scintigraphy didn’t show the accumulation of radiotracer uptake in parathyroid and other parts of the body, thus primary and ectopic hyperparathyroidism was excluded. The kidney function was normal, thus hypercalcemia caused by renal insufficiency was also excluded. Immunofixation electrophoresis and systemic lymph node ultrasonography were normal, tumor markers were all normal. Therefore, multiple myeloma and tumorous diseases can be excluded.

**Fig. 2:**
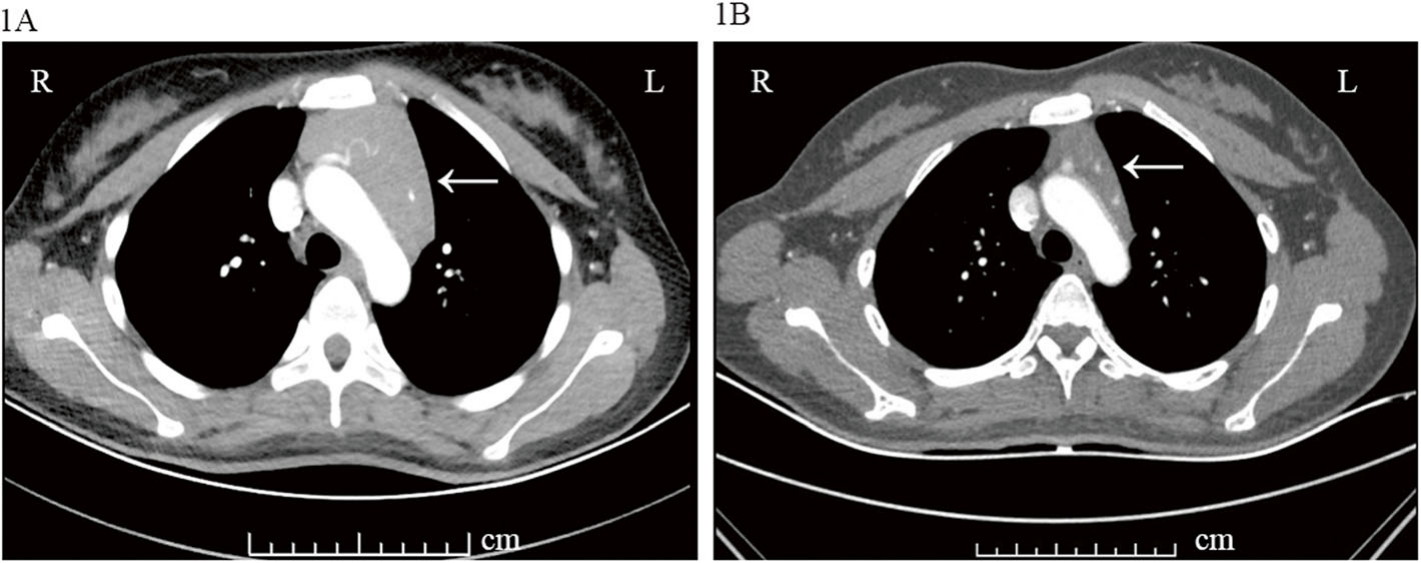
Chest enhanced computer tomography showing patient’s thymus size in relation to the behavior of her GD. **a**: Thymic mass (area at the maximum level: 1963.58mm^2^) at onset of GD (08/ 08/2018); **b**: Significantly reduced thymic mass (area at the maximum level: 911.01mm^2^) after 9 months of treatment with antithyroid drugs ((31/ 05/2019)

However, osteocalcin and beta-CTX were significantly increased (osteocalcin 51.46 ng/mL, normal range: 11–46 ng/ml; beta-CTX 2105.00 ng/L, normal range:< 573 ng/L), and bone mineral density (BMD) demonstrated severe osteoporosis (Z-score: L1–4 BMD − 2.8, Neck BMD − 0.7), which indicated high bone turn-over rate in this patient. Furthermore, the chest enhanced computer tomography (CT) suggested a large mass (1963.58 mm^2^) in the anterior-superior mediastinum (Fig. 2a), and the positron emission tomography computer tomography (PET-CT) also showed this occupation. Although we considered that thymic mass might be the reason for hypercalcemia, surgery was postponed due to uncontrolled hyperthyroidism and hypercalcemia. And when hyperthyroidism was well controlled, the blood calcium decreased gradually, so we continued the follow up of this patient.

After 4 weeks treatment for hyperthyroidism, thyroid function of the patient improved, but did not return to normal, and the blood calcium was restored to the normal range. During the follow-up afterwards, she continued antithyroid treatment, levels of thyroid hormones and TRAb decreased gradually; PTH gradually increased to normal range; blood calcium and phosphorus levels remained in the normal range; and her BMD was also higher than 8 months before (Z-score: L1–4 BMD − 2.4, Neck BMD − 0.7). According to the chest enhanced CT, thymic mass obviously shrank (Fig. 2) 9 months later.

**Fig. 3:**
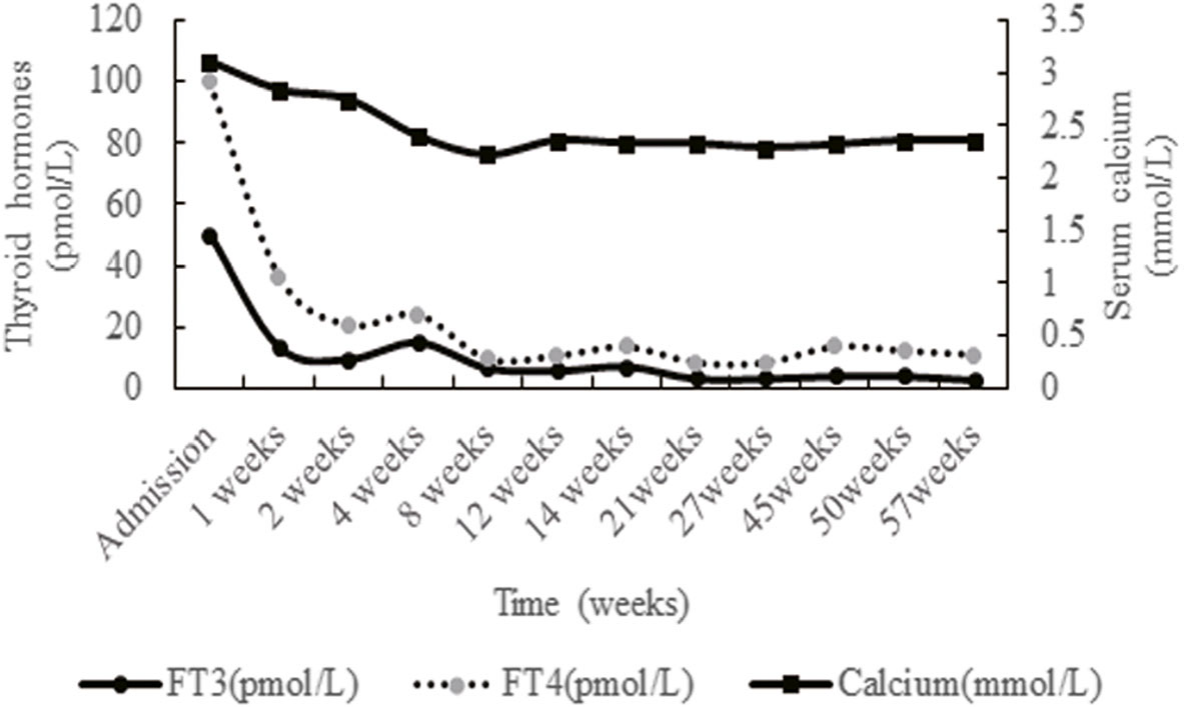
The change of serum calcium and thyroid function for this patient. After 4 weeks treatment for hyperthyroidism, thyroid function improved significantly, blood calcium also decreased. During the follow-up afterwards, levels of thyroid hormones continued to decrease gradually, blood calcium remained in the normal range

After around 14 months of treatment with antithyroid drugs, we tested the thyroid function and TRAb: TSH 3.94 mU/L, FT3 2.79 pmmol/L, FT4 11.0 pmmol/L, and TRAb was 4.57 U/L. for bone turnover markers, beta-CTX was 337.7 ng/L and osteocalcin was 17.35 ng/ml. Blood calcium was 2.36 mmol/L; blood phosphorus was 1.48 mmol/L, and PTH was 30.81 ng/L. BMD also got better and better (Z-score: L1–4 BMD − 2.2, Neck BMD − 0.7). During the whole treatment process, blood calcium gradually decreased and stayed in the normal range after the control of hyperthyroidism (Fig. 3). Furthermore, both FT3 and FT4 levels are negatively correlated with blood calcium level.

## Discussion and conclusions

This is the first case of Graves’ disease accompanied by hypercalcemia, severe osteoporosis and significant enlargement of thymus in a young female, based on the examinations, follow-up of the patient and literature retrieval.

Hypercalcemia, as a common clinical finding with a blood calcium level exceeding the normal range, can expand to many separate diseases with different pathogenesis. It has been reported that nearly 20% of hyperthyroidism patients have coincident mild to moderate hypercalcemia [11], and there are also case reports of hyperthyroidism complicated with hypercalcemic crisis. Thyroid hormones have dual functions on calcium metabolism. On the one hand, they can inhibit the level of active vitamin D in hyperthyroidism patients; the absorption of calcium and phosphorus is decreased in the intestine and kidney, and the excretion of calcium and phosphorus in kidney is increased. On the other hand, thyroid hormones can accelerate the turnover of bone especially osteoclastic activity, which can lead to elevated calcium level. Therefore, a possible mechanism for hypercalcemia in hyperthyroidism might be due to excessive bone absorption induced by elevated thyroid hormones. This patient got elevated serum calcium level in hyperthyroidism condition, and the hypercalcemia was corrected with well control of hyperthyroidism during the follow-up. This result support our primary estimation that hypercalcemia was due to hyperthyroidism in this patient.

Thyroid hormones are critical for bone formation and growth in childhood, and they are also important for adult bone maintenance. In hyperthyroidism adults, thyroid hormones act on bone cells, shorten the re-modelling cycle of bone, resulting in the imbalance between bone resorption and formation, thus causing bone loss and osteoporosis [7]. It is reported that about 5% of secondary osteoporosis had a previous history of hyperthyroidism in a male population [12]. The decline of BMD in hyperthyroid patients has been proved to be reversible after treatment of hyperthyroidism, both bone resorption and absorption markers also declines after an initial rise during subsequent treatment [13]. This patient showed increased bone turnover markers (increased osteocalcin and beta-CTX), and BMD revealed severe osteoporosis, which is in accordance with the influence of thyroid hormones on the bone in previous research given that the patient was young. Consistently, bone turnover markers decreased with improved BMD after the well control of hyperthyroidism.

Thymic mass is a disorder presenting as a mediastinal space-occupying lesion, which mainly occurs in children. In the clinical practice, thymic hyperplasia is often diagnosed incidentally when chest imaging is done for unrelated reasons, and this is becoming more common as chest imaging frequency increases. To our knowledge, Graves’ disease accompanied by thymic hyperplasia was first described in 1912 and confirmed in 1964 [14], but the mechanism behind the coincidence of Graves’ disease and thymic hyperplasia is still uncertified. According to a previous study,^13^ thyroid hormones are associated with thymic size and weight, the thymic volume can be regressed by 61–67% at 6 months after endocrine control. As TSH receptor (TSHR) was expressed in non-neoplastic human thymus, TRAb might induce thymic growth in Graves’ disease. Therefore, the mechanism for the incidence of thymic hyperplasia in Graves’ disease might be the thyroid hormone function and immunologic abnormalities [2]. And for Graves’ disease accompanied by thymic enlargement, only antithyroid treatment and radiological follow-up are warranted [15].

As a previous research revealed that parathyroid hormone-like peptide (PTHrP) receptor is expressed in human thymus [16], so we initially suspected that the thymic mass might secret PTHrP which can result in hypercalcemia. But we could not test PTHrP as we found no commercial test for PTHrP in China. However, During the follow-up, the hypercalcemia was corrected, and the thymus remarkably shrank after the well control of Graves’ disease, which indicated that hyperthyroidism not PTHrP lead to the enlargement of thymus in this patient.

Based on the clinical examinations and related references, we suspected that the hypercalcemia, osteoporosis and thymic enlargement in this patient were all induced by hyperthyroidism, and this speculation was proved by the follow-up results. For Graves’ disease, it is quite rare in that hyperthyroidism cause severe hypercalcemia, osteoporosis and significant thymic enlargement at the same patient. Hyperthyroidism causes calcium and bone metabolism disorders, thus lead to osteoporosis. The thymic enlargement is also induced by elevated thyroid hormones and TRAb levels.

In conclusion, this is a very rare case of Graves’ disease associated with the coexistence of hypercalcemia, severe osteoporosis and significant thymic enlargement. After careful differential diagnosis, a timely control of hyperthyroidism was beneficial for hypercalcemia and osteoporosis, and thymic enlargement is reversible with the antithyroid treatment and does not require surgical intervention. In clinical practice, examination of thymus and bone density should be considered when a patient with Graves’ disease was present with hypercalcemia. Surgery for thymus enlargement in Graves’ disease should be cautious.

## Data Availability

All data generated or analysed during this study are included in this published article.

## Abbreviations

FT3: Free triiodothyronine
FT4: Free thyroxine
TRAb: Anti-thyrotropin- receptor antibody
TSH: Thyroid stimulating hormone
ECT: Emission computerized tomography
PTH: Parathyroid hormone
BMD: Bone mineral density
CT: Computer tomography
PET-CT: Positron emission tomography computer tomography
TSHR: TSH receptor
PTHrP: Parathyroid hormone-like peptide
